# Validity and Reliability of Tamil Translated University of Washington Quality of Life Questionnaire for Head and Neck Cancers

**DOI:** 10.31557/APJCP.2019.20.12.3649

**Published:** 2019

**Authors:** Sivaraman Ganesan, Mahalakshmy Thulasingam, K Gunaseela, R Kalaiarasi, Prasanth Penumadu, Surya Ravichandran, Arun Alexander, Simon N Rogers

**Affiliations:** 1 *Department of ENT, *; 2 *Department of Preventive and Social Medicine, *; 3 *Department of Radiation Oncology, *; 4 *Department of Surgical Oncology, Jawaharlal Institute of Postgraduate Medical Education and Research, Puducherry, India, *; 5 *Regional Maxillofacial Unit, University Hospital Aintree, Liverpool, United Kingdom. *

**Keywords:** World Health Organisation Quality of Life, Head and neck cancer, Pre-treatment, Tamil translation, India

## Abstract

**Background:**

Quality of life is an important outcome measure used both in research and patient care across all cultural healthcare settings.

**Objective:**

This study is aimed to evaluate the validity and reliability of interviewer-administered Tamil translated University of Washington Quality of Life Questionnaire (Version 4) in a setting with low literacy.

**Methods:**

The study was done in a tertiary care teaching institute in Puducherry, South India. The translation was done by using ‘forward-backward translation method.’ A hundred subjects diagnosed with head and neck cancer (HNC) were interviewed before the initiation of treatment. The Tamil version of University of Washington Quality of Life Questionnaire (UWQOL) questionnaire was validated using the Tamil version of the World Health Organization Quality of Life-Brief questionnaire (WHOQOL-BREF) version. Inter-rater reliability and test-retest reliability was also assessed. Item-total correlation and Cronbach alpha were calculated for assessing validity and internal consistency respectively.

**Results:**

In the Tamil version of UWQOL, social-emotional subscale was more affected than physical subscale. The domains such as mood, anxiety, and pain were most affected. The subscale scores were significantly different between those with early and late cancer. Tamil version of UWQOL showed moderate correlation with WHOQOL-BREF. The Tamil version of UWQOL had good test-retest and inter-rater agreement. Item-total correlation for the subscales was >0.80. The internal consistency of the Tamil Questionnaire was acceptable with Cronbach Alpha of 0.69.

**Conclusion:**

The Tamil version of UWQOL questionnaire is a short, valid and reliable for HNC patients of low literacy.

## Introduction

International Agency for Research on Cancer’s online database ‘Global Cancer Incidence Mortality and Prevalence’ (GLOBOCAN) 2018 estimates that the 18.1 million new cancer cases and 9.6 million cancer deaths in 2018 (Bray et al., 2018; International Agency for Research on Cancer, 2018). GLOBOCON 2018 has estimated 1.1 million new cases of cancer in India (GLOBOCAN, 2018). Around 19% of all cancers in India are head and neck cancer (HNC) which include cancer of lip, oral cavity, larynx, oropharynx, nasopharynx, and hypopharynx. In contrast globally, head and neck cancer contribute to 5% of all cancers. However, these cancers are treated with guidelines derived mostly from research findings from the Western world. Hence, the Oncology societies in India recommend researchers to generate evidence for the management of head and neck cancer in the Indian context and also encourage the use of health related quality of life as an outcome measure (Shah et al., 2016).

Health related quality of life is an important outcome as it is recognised that HNC affects the vital function of the subjects (Vartanian et al., 2017). Hence, patient’s subjective experience should reflect the impact of the disease and treatment and this information inform clinical trials and the various cancer management options. Health related quality of life measures physical, emotional, functional and social domains (Catt et al., 2017; Osoba, 2011; D’cruz et al., 2007). In addition, incorporating assessment of quality of life of patients in routine care improves quality of cancer care (Vartanian et al., 2017). 

Multiple general and specific quality of life tools are available (Handle On QOL, 2015). The commonly used scales for head and neck cancers are European Organization for Research and Treatment of Cancer’s Quality of Life Questionnaire, the Functional Assessment of Cancer Therapy scale and the University of Washington Quality of Life questionnaire (UWQOL) (D’cruz et al., 2007). Many of these scales are lengthy and time-consuming to administer especially when used among subjects with low literacy. A scale has also been developed in Indian context considering the socio-cultural and linguistic factors that influence quality of life of cancer patients, however it is not specific to HNC (Vidhubala et al., 2005). The UWQOLv4 is brief with 12 questions (Rogers et al., 2002) and has been validated in India in Hindi and Marathi. It is used by many centers to measure the Health Related Quality of Life (HRQOL) in HNC as it is concise, practical and well validated (Handle On QOL, 2015; Hassan and Weymuller, 1993). A systematic review also identified that UWQOL better measures the impact of appearance issues in patients with HNC (Djan and Penington, 2013)). We translated UWQOL into the Tamil language. Globally, Tamil is spoken by 77 million people and is the official language of Tamil Nadu (India), Singapore and Sri Lanka (Top 30 Language Spoken in the World by Number of Speakers, 2004). In India, Tamil is the spoken language in Tamil Nadu, a South Indian State and Puducherry, a Union Territory of India. According to 2011 census of India, Tamil is the mother tongue of around 6% of Indians and is the fifth common mother tongue (Office of the Registrar General of India, 2018).

This study aims to evaluate the validity and reliability of interviewer-administered Tamil translated version of the UW-QOL (Version 4) in a setting with low literacy. The tool was validated using the World Health Organization Quality of Life Questionnaire BREF (WHOQOL-BREF) version (WHOQOL-BREF, 1996). WHOQOL-BREF was selected as validated Tamil version of the questionnaire was available and is widely used in many situations. 

## Materials and Methods

*Study setting*

The study was undertaken in a tertiary care teaching institute in Puducherry, South India. The hospital has 1,840 beds with about 7,230 patients receiving health care from the institute in a day. Most services are free of cost and are mostly availed by people of low socio-economic status. The hospital based cancer registry functioning at Regional Cancer Centre of the Institute registers on an average of 49 patients with head and neck cancer per month from Regional Cancer Centre attached to the institute. It includes malignancy of oral cavity, pharynx, and larynx (Regional Cancer centre, JIPMER, 2018). 

*UWQOL Questionnaire*

University of Washington Quality of Life Questionnaire version 4 measures health-related quality of life of patients with head and neck cancer. There are 12 domains: pain, appearance, activity, recreation, swallowing, chewing, speech, shoulder function, taste, saliva, mood, and anxiety. Each domain has one question, and the patient responses are scaled from 0 (worst) to 100 (best). It has two sub-scales physical and social function (Rogers and Lowe, 2010). 

*Translation process*

The English version of UWQOL was translated to Tamil by ‘forward-backward translation method.’ The English version was translated to Tamil version by a bilingual person who was Tamil literature teacher. He was briefed about the purpose of the questionnaire. The translated questionnaire was reviewed by two bilingual persons – one ENT doctor and one social worker. They reviewed the Tamil version in view of the English version. They examined and edited the questionnaire for clarity, cultural acceptability, commonly used language by subjects. The edited Tamil questionnaire was back-translated to English by a nurse who was proficient in English. The English and Tamil versions were reviewed and edited by two ENT doctors, a social worker and a public health specialist for conceptual equivalence with the English version. The final Tamil questionnaire was interviewer administered to five patients. Their comprehension of the terms was assessed and was found good. This final questionnaire was used for further data collection. 

*Study Population*

Patients more than 18 years of age, with Tamil as their mother tongue, diagnosed with head and neck cancer seeking care from the tumour clinic conducted by Department of Ear, Nose, and Throat were included in the study. Subjects were enrolled in the study before the initiation of chemotherapy or radiotherapy or surgical treatment for cancer. Patients who were critically ill requiring emergency care were excluded from the study. It is noted that validity measures of rating scales are stable at sample size more than 80 (Hobart et al., 2012). Hence in this study, we included a sample size of 100 subjects. 

*Procedure*

The enrolled subjects were interviewed using a questionnaire. Privacy was maintained during the interview. The questionnaire included questions on socio-demographic profile of the patient, Tamil version of UW-QOL questionnaire and World Health Organization Quality of Life questionnaire BREF Tamil version (WHOQOL-BREF). Details on disease and treatment were collected from the hospital records. American Joint Committee of Cancer staging was followed in the institute to stage cancers. The interview was done by two trained interviewers. Construct validity was assessed by comparing UW-QOL scores with that of WHOQOL-BREF Tamil version. WHOQOL-BREF was chosen as it was validated in a similar culture setting. WHOQOL-BREF has 26 questions. WHOQOL has four domains namely physical health, psychological, social relationships and environment (WHOQOL-BREF, 1996). Subjects were interviewed using the UWQOL thrice to assess test-retest reliability and inter-rater reliability. The first interview was done by interviewer-1 at enrolment, the second interview was done six hours later by interviewer-2 and the third one was done the next day by interviewer-1. To measure test-retest reliability, one-day interval was chosen so that subjects forget the responses they had given, without any clinically meaningful change in the QOL. 

*Statistics*

Data was entered and analyzed in EpiData database (EpiData Association, 2004) and analyzed using EpiData analysis software (EpiData Association, 2004). Sociodemographic and clinical characteristics were summarised as frequency and proportions. The scores of UWQOL was analyzed as per the guidelines provided with the scale (Lowe and Rogers, 2012). Median scores along with 95% confidence interval (95% CI) of the individual items, subscale are reported. Discriminant Validity was assessed by comparing the score of subjects having an early stage with those having late stages of cancer using Mann-Whitney’s test of significance. Construct validity was assessed by comparing UW-QOL scale scores and WHOQOL-BREF scores by Spearman’s correlation. The physical subscale of UWQOL was correlated with the physical domain of WHOQOL- BREF. The socio-emotional subscale of UWQOL was correlated with the psychological domain of WHOQOL-BREF. Inter-rater reliability and test-retest reliability was assessed using Kappa statistics along with 95% confidence interval. Weighted kappa was also calculated using linear weights. Kappa statistics was calculated using GraphPad online tool (GraphPad, 2019). The linear weights were derived using the formula, w_i_=1-i/(k-1), wi indicates the weight for the items for whom the rater differ by i categories and k indicates total number of categories. Based on kappa statistics the strength of agreement was categorised as poor (<0.20), fair (0.21-0.40), moderate (0.41-0.60), good (0.61-0.80), very good (0.81-1.00). Internal consistency of the scale was assessed by (i) item-total correlation and (ii) Cronbach alpha. The p-value <0.05 was considered as statistically significant. 

*Ethical consideration*

The study was approved by the Institute Scientific Advisory Committee and Ethics Committee of JIPMER. Subjects were interviewed after obtaining written informed consent from them. 

## Results

*Socio-demographic and clinical characteristics of study participants*

A total of 100 subjects with head and neck cancer were recruited for the study. The mean age of the subjects was 57 years (SD:10.7). The majority were males (72%), 49% were illiterate and did not receive any formal education. Around 50% of them used tobacco or alcohol for more than a month (Table 1). The major site of malignancy was oral cavity (54%), 81% were in stage III or IV, and 53% had well-differentiated squamous cell cancer (Table 1).

*Domain, subscale and total score of UWQOL*

Table 2 presents the median (IQR) of the domains, subscales and total score of the UWQOL. Mood, anxiety, and pain are the most affected domains. The least affected domains are saliva, taste, speech and shoulder. Social-emotional function subscale is more affected than the physical functions. In the general questions of UWQOL, 55 and 29 subjects rated poor/very poor for their health-related quality of life and overall quality of life respectively. 

*Validity measures*

The Tamil version of UWQOL was compared with the scores obtained by the subjects in the Tamil version of WHOQOL-BREF. The UWQOL total score had significant positive correlation with WHOQOL total score (r=0.6). Moderate correlation was also observed in the subscales of physical (r=0.49) and psychological (r=0.56) subscale of WHOQOL. Discriminant validity was assessed by comparing the scores between those with early and late stage of cancer. Subjects with advanced cancer stage obtained a significantly worse scores in the physical and social-emotional subscales. Those with advanced cancer stage scored lower in most of the domains, especially pain, activity, swallowing and chewing (Table 2). 

*Reliability measures*

The results of test-retest and inter-rater agreement of the questionnaire using Kappa Statistics is given in Table 3. For one-day interval test-retest reliability, almost all items in the Tamil version of UWQOL had good agreement (kappa =0.61 to 0.80). Pain and taste had moderate agreement. With regards to inter-rater agreement all items except mood and anxiety had good agreement. 

*Internal Consistency*

Item to total score correlation of the domains ranged from 0.15 to 0.75. The subscale to total score correlations were high. Tamil version of UWQOL scale has acceptable internal consistency.

**Table 1:** Socio-demographic and Clinical Characteristics of Study Participants

Characteristics	Frequency
Total	100
Age in years, mean (SD)	57.2 (10.7)
Gender	
Male	72
Female	28
Education	
Illiterate	49
1-10 years of formal education	36
> 10 years of formal education	15
Ever tobacco smokers^a^	57
Ever users of smokeless tobacco^a^	43
Ever alcohol users^a^	58
Primary site of malignancy	
Oral cavity	54
Oro Pharynx	12
Laryngo pharynx	34
Histopathology of malignancy	
Well differentiated SCC	53
Moderately differentiated SCC	31
Poorly differentiated SCC	5
Not recorded	11
Cancer stage	
I	6
II	13
III	24
IV	57

^a^Ever users, Use of tobacco/alcohol for more than one month including current users; SCC, Squamous Cell Cancer

**Table 2:** Tamil Version of UWQOL Scores Obtained by the Study Participants, n=100

Variables	All subjects	Early Cancer stage, n=19^c^	Late cancer stage, n=81^c^	Item Total Correlation^d^
	Median (IQR)	Median (IQR)	Median (IQR)	
Items				
Pain	50 (50-69)	50 (50-100)*	50 (50-50)*	0.46
Appearance	75 (75-100)	75 (75-100)*	75 (75-75)*	0.43
Activity	75 (0-100)	100 (75-100)*	50 (0-100)*	0.75
Recreation	75 (50-100)	75 (50-100)*	75 (50-100)*	0.59
Swallowing	70 (30-100)	100 (70-100)*	70 (30-100)*	0.58
Chewing	75 (0-100)	100 (100-100)*	50 (0-100)*	0.51
Speech	100 (30-100)	100 (100-100)*	100 (30-100)*	0.50
Shoulder	100 (30-100)	100 (70-100)*	100 (30-100)*	0.38
Taste	100 (100-100)	100 (100-100)*	100 (100-100)*	0.30
Saliva	100 (100-100)	100 (100-100)*	100 (100-100)*	0.15
Mood	25 (25-50)	50 (25-75)*	25 (25-50)*	0.51
Anxiety	30 (30-70)	30 (30-70)*	30 (30-70)*	0.42
Subscales				
Physical^a^	75 (63-91)	84 (72-96)*	72 (62-88)*	0.84
Social-emotional^b^	56 (40-68)	68 (55-74)*	51 (38-68)*	0.86

^a^Physical function subscale includes items on chewing, swallowing, speech, taste, saliva, appearance; ^b^Social-Emotional function subscale includes items on anxiety, mood, pain, activity, recreation, shoulder function; ^c^early cancer stage includes stage I or II, late cancer stage includes stage III or IV; ^d^Spearman correlation coefficient between the scores in each item and the total score; * p value <0.05 using Mann Whitney test of significance

**Table 3:** Reliability Measures of Tamil Version of UWQOL, n=100

Items^a^	Test-Retest			Inter-Rater		
	Kappa	(95% CI)	Weighted Kappa^b^	Kappa	(95% CI)	Weighted Kappa^b^
Pain	0.56	(0.42-0.69)	0.55	0.62	(0.49-0.76)	0.68
Appearance	0.76	(0.65-0.88)	0.77	0.62	(0.49-0.75)	0.63
Activity	0.77	(0.68-0.87)	0.83	0.65	(0.54-0.76)	0.77
Recreation	0.68	(0.56-0.81)	0.68	0.62	(0.49-0.75)	0.64
Swallowing	0.67	(0.56-0.79)	0.76	0.66	(0.54-0.78)	0.72
Chewing	0.77	(0.66-0.88)	0.84	0.64	(0.52-0.77)	0.69
Speech	0.79	(0.68-0.90)	0.82	0.66	(0.53-0.79)	0.76
Shoulder	0.79	(0.69-0.91)	0.81	0.69	(0.56-0.82)	0.73
Taste	0.53	(0.33-0.74)	0.54	0.68	(0.50-0.86)	0.67
Mood	0.69	(0.56-0.80)	0.70	0.44	(0.31-0.58)	0.45
Anxiety	0.60	(0.45-0.76)	0.62	0.36	(0.21-0.52)	0.44

^a^Kappa was not calculated for the item saliva as 99 subjects out of 100 included in the study did not have symptoms related to salivation as the subjects were recruited before initiation of treatment. ^b^Weighted Kappa was calculated using the linear weights.

## Discussion

The study comprised 100 subjects with HNC prior to the treatment. Majority of the participants were males, similar to the epidemiology of head and neck cancer in India. In India the age standardized incidence head and neck cancers are around three times higher in males (International Agency for Research on Cancer, 2018). Measuring pre-treatment HRQOL is essential as this can be used to monitor change over time. In the UWQOL, social-emotional subscale was more affected in particular the domains of mood, anxiety and pain. The subscale scores were significantly different between those with early and late cancer. Tamil version of UWQOL showed moderate correlation with WHOQOL-BREF. The Tamil version of UWQOL had good test-retest reliability, inter-rater reliability, item-total correlation, and acceptable Cronbach Alpha. 

Head and Neck cancer and its treatment impact multiple spheres of a person’s life (Taylor et al., 2004; Vartanian et al., 2006). Hence, recently the HRQOL assessment is recommended in cancer-related researches (Hassan and Weymuller, 1993; José Guilherme Vartanian et al., 2004; Murphy, 2009; Rogers and Barber, 2017; Vartanian et al., 2017) and in patient care setting (Rogers et al., 2002). The study participants had low literacy and were from lower socio-economic status. In subjects with low literacy, the questionnaire needs to be administered by an interviewer or should be assisted by technology (Hahn and Cella, 2003). Interviews are feasible when the questionnaire is quick, straightforward to use and suitable for a busy clinical setting. UWQOL has these characteristics, hence more practical and cost-effective (Nazar et al., 2013; Rogers et al., 2002). Thereby, it suits quality of life assessment in low literate population. The UWQOL has been translated and validated in various languages (Merseyside Regional Head and Neck Cancer, 2019) in Hindi, Marathi (D’cruz et al., 2007), Brazilian (Andrade et al., 2012), Brazilian-Portuguese (Jose Guilherme Vartanian et al., 2006), Chinese (Lee et al., 2017), Spanish (Nazar et al., 2013), Greek (Linardoutsos et al., 2014), Turkish (Şenkal et al., 2012). In this project, we translated and validated UWQOL in the Tamil language. Since the questionnaire was validated in low literate population, the questionnaire was interviewer administered, and we measured inter-rater reliability. 

For assessing construct validity, we compared the UWQOL score with WHOQOL-BREF. There was a moderate correlation between the two scores. Similar, observations were noted in others studies which used general questionnaire instead of disease-specific questionnaire (Vartanian et al., 2006; Nazar et al., 2013; Şenkal et al., 2012). The total score on UWQOL was significantly different between the early and late stage of cancer. Similar results were noted by other studies (Lee et al., 2017; Linardoutsos et al., 2014; Nazar et al., 2013; Şenkal et al., 2012). The overall mean score observed in the study was lower than that observed in other studies (Adnane et al., 2016; Jose Guilherme Vartanian et al., 2006; Lee et al., 2017; Linardoutsos et al., 2014; Nazar et al., 2013; Sakthivel et al., 2017; Şenkal et al., 2012). The subjects were interviewed before initiation of treatment. It could also be attributed to a high percentage (81%) of our subjects were in the late stages of cancer. This distribution mimics the cancer epidemiology in India where 60-80% present at the late stage of the disease (Kulkarni, 2013).

The Tamil version of UWQOL was also stable with good test-retest reliability as noted by other versions (Lee et al., 2017; Şenkal et al., 2012). The internal consistency assessed by Cronbach’s α coefficient was acceptable (0.7); similarly, item-total correlation was good as noted in other studies (D’cruz et al., 2007; Jose Guilherme Vartanian et al., 2006; Şenkal et al., 2012). The English (Rogers et al., 2002) and the Greek (Linardoutsos et al., 2014) versions of the questionnaires had higher internal consistency. 

The study was restricted to pre-treatment patients, and further studies on post-treatment and longitudinal follow-up evaluating outcome are required. Adequate sample size, testing of questionnaire in a low literate group, calculation of inter-rater reliability and comparison with WHOQOL-BREF which is well validated in Tamil were the strengths of the study. The Tamil version of UWQOL questionnaire was valid and reliable when administered by interviewer among patient with low literacy. 
